# Interprofessional competencies: the poor cousin to clinical skills?

**DOI:** 10.15694/mep.2017.000123

**Published:** 2017-07-07

**Authors:** Priya Martin, Monica Moran, Dawn Forman

**Affiliations:** 1 Darling Downs Hospital and Health Service; 2 The University of Western Australia; 3 Interactive Leadership and Management Development

**Keywords:** Interprofessional Education

## Abstract

This article was migrated. The article was marked as recommended.

The purpose of this paper is to clarify what work-based IPE is, challenge some common misconceptions about its values in clinical settings and highlight tools that will assist with its implementation in such settings.

## Interprofessional competencies: the poor cousin to clinical skills?

It is all too common in health settings where students receive practice education to come across misconceptions amongst health professionals about what interprofessional education (IPE) is and what strategies are involved in its delivery. In our (the authors) roles as clinical educators and health professional education researchers, we often hear clinician colleagues describe interprofessional competencies as “soft skills” and see them as the “poor cousin” to clinical skills. We believe that these misconceptions arise from a lack of understanding of what IPE in the work setting looks like, its contribution to the development of interprofessional competencies and ultimately its value in improving health outcomes. The purpose of this paper is to clarify what work-based IPE is, challenge some common misconceptions about its values in clinical settings and highlight tools that will assist with its implementation in such settings.

It is well-accepted that IPE occurs when students or members of two or more professions learn with, from and about each other to improve collaboration and the quality of patient care ( Barr & Lowe 2013). Most ongoing interprofessional learning is work-based ( Barr & Lowe 2013). Work-based IPE provides opportunities for students to compare professional perspectives, share knowledge, learn about other’s roles and responsibilities, and explore ways to collaborate more closely within a fluctuating real world health environment ( Barr & Lowe 2013). It is desirable to facilitate IPE as early as possible in the pre-registration stage (i.e., before graduation) as students are still forming beliefs and attitudes related to healthcare practice ( WHO 2010). Ideally, interprofessional education in student placements would involve students learning from each other. For example, a medical student and a physiotherapy student completing a placement on an acute ward simultaneously have the chance to learn about each other’s roles if opportunities are created.

We agree with Nicol and Forman’s (2014) descriptions of the attributes necessary for effective interprofessional education placements:

• Have relevance to the individual discipline
• Have individual discipline support for the student
• Have a trained interprofessional facilitator
• Have staff who were acquainted with interprofessional learning and where necessary adaptations are made to support this sort of learning and
• Ensure students are prepared appropriately for this sort of learning.

We believe that misconceptions about IPE arise from a number of sources. Firstly a lack of knowledge about interprofessional practice can generate negative attitudes towards educating students in this way. We also opine that working in professional silos, competition between professions and tribalism of professions contribute to these negative attitudes. The hidden curriculum of unspoken or implicit values, behaviours, procedures and norms particularly around professional status in the health setting can also hinder IPE and impact on collaboration with other team members and at worst leave health professionals fearful about threats to their roles. The intention of IPE is not role substitution or dilution of skills, or generalistion of the health workforce. From an organisational point of view a common misconception is the belief that a large number of students from many professions are required to facilitate IPE. However, in practice we have observed IPE being facilitated with a minimum of two students from two professions. Finally a serious concern as we have already alluded is that interprofessional competencies such as interprofessional collaboration and conflict resolution are seen as less essential skills then specific clinical skills, despite the fact that many adverse events in health settings are linked back to poor communication or information sharing across the professions involved.

We like tools such as the Canadian national interprofessional competency framework ( CIHC 2010) and the Framework for Action on Interprofessional Education and Collaborative Practice World Health Organisation ( WHO 2010) as they provide explicit guidelines to establish or support work-based IPE. The Canadian framework outlines six competency domains namely interprofessional communication, patient/client/family/community-centred care, role clarification, team functioning, collaborative leadership and interprofessional conflict resolution that can be embedded in work based interprofessional learning environments. This framework allows users to learn and apply the competencies no matter their level of skill or type of practice setting or context ( CIHC 2010). An important feature of this framework is the central inclusion of the patient/family/community as part of the interprofessional healthcare team ( CIHC 2010). The WHO Framework identifies the mechanisms that shape successful collaborative teamwork and outlines a series of action items that policy-makers can apply within their local health system to incorporate IPE. Furthermore, it outlines a number of educator mechanisms (such as staff training, identification of IPE champions, institutional and managerial commitment and identification of learning outcomes) and curricular mechanisms (such as logistics and scheduling, shared objectives, learning methods, adult learning principles, contextual learning and assessment) to integrate IPE in practice ( WHO 2010).

We agree with Hean and colleagues (2013), who argue that educators in addition to using IPE frameworks need to explore the theories that psychosocial and related disciplines offer. They assert that theories such as social capital, social constructivism and sociology’s scepticism facilitate IPE through building social relationships between learners and teaching staff, as well as enable staff to transition from independent work underpinned by their own professional knowledge to collaborative working.

In summary, the benefits of IPE in growing interprofessional competencies and maximising health outcomes have been established internationally ( WHO 2010). Whilst the benefits of work-based IPE are wide-ranging (e.g., better patient outcomes, efficient use of resources, improved coordination of patient care, avoidance of duplication), we have outlined some deterrents that inhibit its implementation in the health setting. We believe that time is ripe to openly address these barriers and educate health practitioners on the value of work-based IPE and the strategies available to assist with this process. Given global workforce issues such as shortage of health workers and a growing constraint on health resources ( WHO 2010) we think that it is essential for health practitioners and educators to embrace IPE and facilitate its implementation in the health setting. We maintain that IPE is no longer an optional strategy as IP competencies are integral in achieving a collaborative practice-ready health workforce ( CIHC 2010; WHO 2010). Therefore, it is more imperative than ever to identify and address the barriers to IPE in the health setting, to challenge misconceptions and move away from a faulty dichotomy of hard clinical skills versus soft interprofessional skills.

## Take Home Messages

• The benefits of IPE in growing interprofessional competencies and maximising health outcomes have been established internationally.
• Time is ripe to openly address the barriers in implementing IPE and educate health practitioners on the value of work-based IPE and the strategies available to assist with this process.

## Notes On Contributors

Ms Martin is Advanced Clinical Educator in Queensland, Australia and PhD Candidate at the University of South Australia

Dr Moran is Associate Professor of Rural Health at the University of Western Australia.

Dr Forman is Director of Interactive Leadership and Managemnet Development and Visiting Professor at the University of Derby and Chichester University, United Kingdom.
