# Supplementary material for: Interprofessional competencies: the poor cousin to clinical skills?
Source: MedEdPublish (2016). 2017 Jul 7;6:123. [Version 1] doi: 10.15694/mep.2017.000123 (PMC10885276; doi:10.15694/mep.2017.000123)
Supplement: Peer review history

# Reviewer report: Reviewer response for version 1

**DOI:** 10.21956/mep.19314.r28180

**Published:** 2017-07-12

**Authors:** Susan Van Schalkwyk

**Affiliations:** 1 Stellenbosch University

This review has been migrated. The reviewer awarded 4 stars out of 5 This opinion piece seeks to counter some of the misconceptions, as perceived by the authors, relating to IPE – both in terms of what it is and how it should be delivered. I would agree with the authors that misconceptions do exist, although I suspect that given the growing body of literature in this area, the prevalence of such misconceptions could be diminishing. However, whether these misconceptions lead to them being regarded as ‘soft skills’, is probably up for debate. The piece could therefore dealing with two separate issues that require two different responses – the one to seek to address misconceptions about what it is, the other to shift thinking in terms of ‘soft skills’ and thus being seen as ‘the poor cousin’. If one accepts this view then the title may not quite reflect the content in the piece.Having said that, I appreciated the fact that the authors have drawn on their personal experience to foreground an important issue and open it up for debate. Further reference to current literature could have strengthened their position.

Reviewer Expertise:

NA

No decision status is available

# Reviewer report: Reviewer response for version 1

**DOI:** 10.21956/mep.19314.r28181

**Published:** 2017-07-11

**Authors:** James Fraser

**Affiliations:** 1 Griffith University

This review has been migrated. The reviewer awarded 4 stars out of 5 In workplace base learning it can be easy to assume that IPE occurs as a natural result of students immersing themselves in the clinical environment and this paper reminds us that challenges still remain in this important curricula area. Facilitating developmentally appropriate and authentic IPE opportunities for students in a busy curriculum will require faculty development for the clinician educators and clear alignment of equivalent learning objectives for the students from the professions involved. Assessing and evaluating the short-term impacts of IPE may require us to consider innovative methods to provide feedback to the students to help them plan for future development. A clear demonstration of the long term impact of patient safety and clinical outcomes may help convince the slow adopters of the importance of IPE in health professional training.

Reviewer Expertise:

NA

No decision status is available

# Reviewer report: Reviewer response for version 1

**DOI:** 10.21956/mep.19314.r28182

**Published:** 2017-07-10

**Authors:** Nandalal Gunaratne

**Affiliations:** 1 Faculty of Medicine, Wayamba University of Sri Lanka

This review has been migrated. The reviewer awarded 4 stars out of 5 The barriers include lack of interest by parties concerned to be educated this way. To be successfully initiated this is the first thing to remove. Secondly should it be initiated by the "top" or "bottom" in the professional hierarchy?

Reviewer Expertise:

NA

No decision status is available

# Reviewer report: Reviewer response for version 1

**DOI:** 10.21956/mep.19314.r28183

**Published:** 2017-07-07

**Authors:** Trevor Gibbs

**Affiliations:** 1 AMEE

This review has been migrated. The reviewer awarded 4 stars out of 5 An interesting and important commentary on a very important subject. I felt that it was very necessary to reflect these feelings that still exist, although there are many papers that show the development of IPE remains as strong as ever. As someone who travels quite extensively to developing countries, perhaps I would have liked to have seen some commentary upon how IPE still remains an initiative amongst the developed countries; whereas development of IPE in less well-developed countries could have a profound effect upon healthcare.I do also think that we need to be looking further- what are the longer term implications of IPEI am sure that this paper will raise further interest and discussion

Reviewer Expertise:

NA

No decision status is available
